# Integrating Ultimate and Proximate Explanations of Neurodivergence: Evolutionary Trade‐Offs, Compensation, and Clinical Expression

**DOI:** 10.1111/eva.70284

**Published:** 2026-06-18

**Authors:** Darko Sarovic

**Affiliations:** ^1^ Institute of Clinical Sciences University of Gothenburg Gothenburg Sweden; ^2^ Paediatric Health Professions and Paediatric Radiology Sahlgrenska University Hospital Gothenburg Sweden; ^3^ Department of Women's and Children's Health Uppsala University Uppsala Sweden

**Keywords:** adaptive capacity, autism, evolutionary psychiatry, neurodivergence, pathogenetic triad, ultimate and proximate causation

## Abstract

Elevated rates of major depressive disorder and anxiety disorders in neurodivergent populations are often interpreted as evidence that autism, attention‐deficit/hyperactivity disorder, and affective disorders share intrinsic neurobiological pathology. Evolutionary psychiatry offers a different interpretation: neurodivergent trait profiles may reflect evolutionarily shaped cognitive variation whose consequences depend on ecological and social fit. Recent evolutionary psychiatry work has extended this perspective by interpreting the concomitance of neurodivergence with depression and anxiety through evolutionary trade‐off, mismatch, and evolved affective defense systems. Here, this ultimate‐level account is integrated with the pathogenetic triad, an operationalized cross‐level framework that adds an individual‐level account of how traits, adaptive capacity, burden, and environmental demands shape clinical expression. This synthesis suggests one pathway by which depression and anxiety may arise in neurodivergent individuals: chronic mismatch may increase compensatory demands and repeatedly activate evolved affective and defensive systems, particularly when regulatory buffering is limited or burden is high. The resulting framework helps reconcile neurodiversity and medical models by distinguishing natural neurodivergent trait variation from the mechanisms through which it becomes clinically impairing. Evolutionary psychiatry helps explain why neurodivergent traits persist and why mismatch may generate vulnerability; the pathogenetic triad helps specify how, when, and for whom such vulnerability becomes clinically expressed.

## 1 Evolutionary Psychiatry and the Problem of “Comorbidity”

Elevated rates of major depressive disorder and anxiety disorders in neurodivergent populations are often interpreted as evidence that autism, attention‐deficit/hyperactivity disorder (ADHD), and affective disorders share underlying neurobiological pathology. An alternative interpretation is that neurodivergent trait profiles may represent evolutionarily shaped cognitive variation whose consequences depend on ecological and social fit. Earlier evolutionary psychiatry work has already argued that ADHD‐related traits may be partly understood through evolutionary mismatch, trade‐offs, and environmental fit, particularly in relation to modern schooling and occupational demands (Swanepoel et al. 2017), while later work has extended similar reasoning to both autism and ADHD as forms of biological variation that are not inherently disordered in all cases (Swanepoel 2024; Swanepoel et al. 2024). Griffin et al. (2026) extend this literature by applying trade‐off and mismatch reasoning to the elevated concomitance of neurodivergence with major depressive disorder and anxiety disorders.

This reframing is valuable because biomedical, neurodiversity, and social accounts each capture part of the phenomenon but become incomplete when treated as sufficient on their own. Biomedical accounts correctly emphasize impairment, developmental burden, treatment need, and the high concentration of biological and psychosocial risk in clinically ascertained populations but may overextend this logic by treating the neurodivergent trait profile itself as intrinsically pathological. Neurodiversity and social accounts correctly emphasize cognitive variation, stigma, barriers, and mismatch, but may understate the developmental, regulatory, cognitive, and psychosocial constraints that shape compensation and clinical outcome. An evolutionary account helps avoid this dichotomy by treating neurodivergence as cognitive diversity whose expression depends on the interaction with adaptive capacity, burden, and environment.

This is precisely the kind of question for which evolutionary psychiatry is well suited. It asks why certain traits persist, why they may have been maintained by selection or balancing mechanisms, and why evolved systems that were adaptive in ancestral or specific ecological contexts may become costly under modern environmental conditions. However, ultimate explanations do not by themselves specify why some individuals with neurodivergent traits remain adaptive, subclinical, or self‐identified, whereas others receive clinical diagnoses and experience substantial impairment and broader psychiatric or somatic burden.

The present article extends this synthesis by integrating the evolutionary account of neurodivergence‐environment mismatch with the pathogenetic triad, a cross‐level framework developed to explain how neurodevelopmental trait variation becomes clinically expressed (Sarovic 2019, 2021). In brief, the pathogenetic triad proposes that clinical neurodevelopmental expression depends on the interaction among three partially separable components: a phenotype‐specific neurodivergent trait profile, Cognitive Capacity, and Neuropathological Burden. The trait profile specifies the kind of neurodevelopmental phenotype being expressed, such as autistic or ADHD‐related traits. Cognitive Capacity refers to the cognitive and executive resources that allow the individual to compensate for difficulties arising from this profile. Neuropathological Burden refers to the developmental, biological, physiological, and psychosocial constraints that may disrupt neurodevelopment, reduce regulatory stability, or limit compensatory capacity. In this view, diagnosis does not reflect trait magnitude alone, but the point at which trait expression, compensation, burden, and environmental demands interact to produce persistent maladaptation. The triad already contains an evolutionary premise: neurodivergent trait profiles, cognitive compensatory abilities, and immune‐autonomic regulatory buffering are not merely proximate mechanisms, but evolutionarily shaped traits and systems. Its distinctive contribution is to specify the individual‐level architecture through which such systems interact with burden and environmental demands to produce adaptive, subclinical, decompensating, or diagnosable outcomes. The specific aim of the present Perspective is therefore to translate Griffin et al.'s (2026) ultimate‐level account of trade‐offs, mismatch, and affective defense activation into a clinical framework for understanding how, when, and for whom neurodivergent vulnerability becomes expressed as impairment, anxiety, depression, or broader clinical burden.

Two boundaries should be made explicit before developing the argument further. First, neurodivergence is used here primarily to refer to autism‐ and ADHD‐related neurodevelopmental trait profiles; depression and anxiety are treated as affective outcomes or concomitant conditions, not as neurodivergent traits themselves. Compensation denotes cognitive, behavioral, or environmental strategies that support functioning without eliminating or normalizing the underlying trait profile, whereas burden denotes the developmental, biological, physiological, or psychosocial constraints on regulatory stability or adaptive capacity, not distress, impairment, or diagnosis itself. Second, because this is a conceptual Perspective, the synthesis is offered as a hypothesis‐generating framework rather than as a claim that the full pathway from mismatch to compensation, regulatory strain, and clinical outcome has already been tested. Although triad‐related postulates and quantitative triad‐space formulations have begun to be tested (Sarovic 2026), full evaluation of the proposed multilevel pathway will require converging evidence across longitudinal, clinical, physiological, developmental, and environmental studies.

## 2 Ultimate and Proximate Levels of Explanation

Mayr's distinction between ultimate and proximate causation remains useful in psychiatry because it prevents different kinds of explanations from being treated as competitors (Mayr 1961; Nesse 2023). Ultimate explanations address why a trait exists or persists: its evolutionary history, adaptive trade‐offs, constraints, and ecological conditions of advantage or disadvantage (Garland Jr. et al. 2022). Proximate explanations address how the trait is experienced and expressed: its developmental, biological, cognitive, physiological, and environmental mechanisms.

Griffin et al. (2026) foreground the ultimate/evolutionary side of this account. Their central contribution is to argue that neurodivergence and affective vulnerability can be interpreted through evolutionary trade‐offs, social niche specialization, environmental mismatch, and evolved affective defense systems, rather than through intrinsic defect. In this account, neurodivergent cognitive profiles may reflect forms of specialization that are beneficial in some niches but become vulnerable in others, such as when contemporary educational, occupational, sensory, and social environments demand conformity to a narrow cognitive norm. Depression and anxiety may then arise not because neurodivergence is itself pathological but because mismatch is cognitively costly, repeatedly activating affective and defensive systems that evolved to respond to threat, uncertainty, exclusion, or unattainable goals. This provides a useful entry point into the broader question of how ultimate explanations of neurodivergent trait persistence can be connected to proximate mechanisms of clinical expression.

The pathogenetic triad adds a complementary layer to this argument. It is not simply the proximate counterpart to Griffin et al.'s (2026) evolutionary account because the triad itself treats the relevant trait profile, compensatory capacity, and burden‐buffering systems as evolved, developmentally shaped systems. Its distinctive contribution is instead architectural: it specifies how these systems interact at the individual level to produce clinical outcomes. In this sense, the triad connects evolutionary claims about trade‐offs and mismatch to clinical heterogeneity, compensation, decompensation, and diagnosis.

**FIGURE 1:**
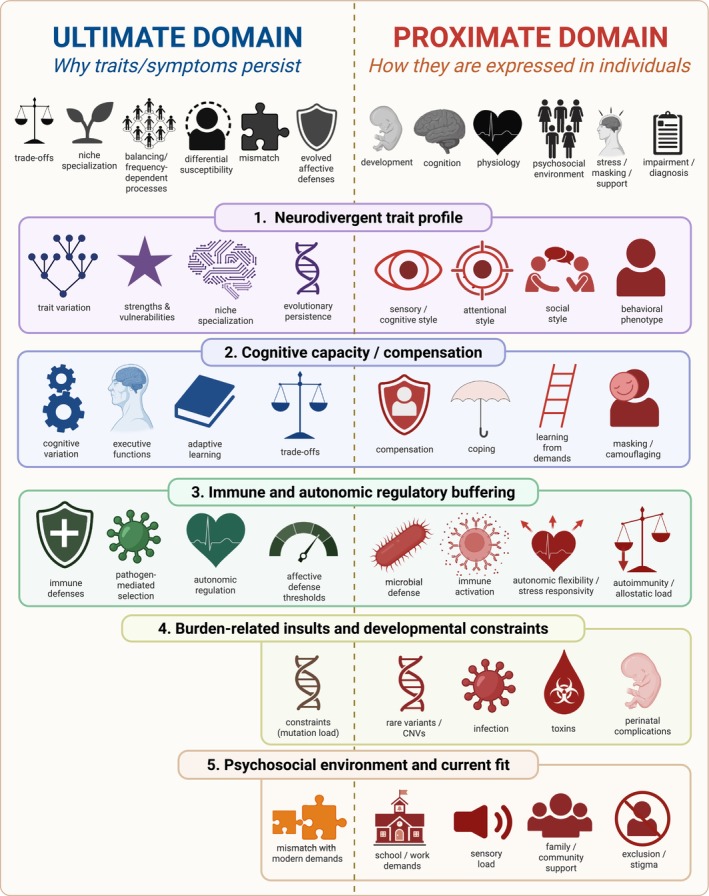
Ultimate and proximate domains in the extended pathogenetic triad framework. The figure maps triad‐related factors onto complementary levels of explanation. The ultimate domain concerns why neurodivergent traits and regulatory systems may persist across populations, including mechanisms of trade‐off, niche specialization, mismatch, and evolved affective activation. The proximate domain concerns how these traits and systems are expressed in individuals through developmental, cognitive, physiological, and psychosocial pathways. Rows 1–5 organize the extended framework into neurodivergent trait profile, cognitive capacity and compensation, immune and autonomic regulatory buffering, burden‐related insults and developmental constraints, and psychosocial environment and current fit. Some components span both domains, linking evolutionary persistence to individual expression, whereas others are more specifically situated within proximate mechanisms. The figure is intended as a conceptual map of explanatory levels; the dynamic interaction among these factors is addressed separately in the triad‐space representation. Created in BioRender; Sarovic (2026), https://BioRender.com/5ogt6h0.

Figure 1 maps this division of labor. Griffin et al. (2026) make the environmental side of the evolutionary account especially explicit by linking mismatch to affective disorders in neurodivergent populations. The triad extends the same evolutionary reasoning to adaptive capacity itself: Cognitive Capacity, autonomic regulation, and immune function are genetically influenced, developmentally plastic systems with potential strengths and liabilities (Korte et al. 2005; Plomin and Deary 2015; Quintana‐Murci 2019). The central point is therefore not that one framework is ultimate and the other merely proximate, but that a complete account must explain both why the neurodivergent traits and regulatory systems persist and how their interaction with burden and environment produces clinical expression.

## 3 A Cross‐Level Architecture for Clinical Expression

The first triad component is the phenotype‐specific neurodivergent trait profile: the neurodevelopmental or neuropsychiatric personality configuration that gives a condition its phenotypic specificity (Sarovic 2019). In this sense, autistic phenotypic expression is defined by autistic traits, ADHD‐related expression by an ADHD‐related trait configuration, and other neurodevelopmental conditions by their corresponding trait profiles. CC, NB, and environmental demands modulate functional expression and clinical salience, but they do not by themselves specify the phenotype.

In the autism‐specific operationalization of the framework, this phenotype‐specific component was termed Autistic Personality (AP), referring to a proposed non‐pathological, dimensional autistic trait configuration related to the broader autism phenotype (Sarovic 2021). The term denotes the autism‐specific form of the first triad component, not neurodivergence as a whole or autism diagnosis itself, and it does not imply pathology or a categorical personality disorder; Box 1 clarifies how AP is used in the present framework.

BOX 1 **Autistic personality, autistic traits, and autism diagnosis**

Autistic Personality (AP) refers to the proposed autism‐specific trait component of the pathogenetic triad. It denotes a dimensional configuration of autistic traits related to the broader autism phenotype, rather than a clinical diagnosis. In this usage, “personality” refers to a stable trait configuration.

AP is conceptually related to autistic traits observed in diagnosed autism, but autism diagnosis is not treated as a direct measure of AP magnitude alone. Diagnosis requires clinically significant impairment and is shaped by compensatory capacity, developmental burden, as well as by clinical ascertainment. Genetic findings support partial continuity between autism liability and population‐level variation in social‐communicative and adaptive traits (Robinson et al. 2016), but clinically diagnosed samples are also enriched for additional sources of impairment. AP should therefore be understood as a proposed dimensional trait substrate, whereas autism diagnosis reflects the clinical expression of that substrate under particular compensatory, burden‐related, and environmental conditions.

Analogous first‐factor constructs could be proposed for other neurodevelopmental profiles, but their content would differ. For example, an ADHD‐related trait configuration would not be AP; it would instead involve traits related to attentional regulation, activity level, reward responsiveness, and exploratory behavior (Barkley 1997; Sonuga‐Barke 2003; Swanepoel et al. 2017; Williams and Taylor 2006).

With this phenotype‐specific component clarified, the remaining triad components explain why similar trait profiles may differ in functional and clinical expression. The second component is CC, understood as one aspect of adaptive capacity. It denotes the cognitive and executive resources that help an individual manage trait‐related demands, such as learning explicit rules, inhibiting maladaptive responses, structuring behavior, and managing sensory or cognitive load. Although intelligence and executive functions are relevant, CC is broader than any single psychometric score, and empirical operationalizations will necessarily capture only parts of the theoretical construct. In the triad, reduced CC does not create the neurodivergent trait profile itself; rather, it narrows the range of contexts in which that profile can remain functional, thereby increasing the likelihood of impairment or diagnostic salience when demands are high.

The third component is NB, conceptualized as the aggregate effect of endogenous and exogenous constraints that can disrupt neurodevelopment, reduce regulatory stability, or limit compensatory capacity. The triad emphasizes that risk factors associated with neurodevelopmental diagnoses are unlikely to be specific components of the neurodivergent core. Instead, they are transdiagnostic risk factors: they increase the probability of maladaptation and may raise the risk for multiple neurodevelopmental, psychiatric, and somatic outcomes, rather than explaining any one diagnosis specifically. This includes genetic syndromes and rare deleterious variants, as well as infectious/immune, neurological, toxic, perinatal, stress‐related, and other exposomic influences that can constrain neurodevelopment or compensation (Modabbernia et al. 2017; Satterstrom et al. 2020; Weiner et al. 2017). Within this broad NB construct, immune and autonomic regulatory systems are treated as burden‐buffering mechanisms whose integrity modulates how strongly such exposures affect neurodevelopment, physiological stability, and cognitive compensation. Here, biopsychosocial exposomic burden denotes the cumulative exposures that constrain neurodevelopment, regulatory stability, or compensatory capacity across the life course, whereas proximal environmental fit refers to the current relation between an individual's trait profile and immediate contextual demands, supports, and accommodations.

This architecture is important because it separates the core trait from the mechanisms that make the trait clinically consequential. The autism‐specific pathogenetic triad paper (Sarovic 2021) illustrated this logic by presenting the broader autism phenotype as a non‐pathological personality domain, shared across the population and largely uncoupled from associated features such as low cognitive ability and immune dysfunction. It also proposed that common genetic variation contributes to the personality domain, whereas rare deleterious variants and other risk factors may act partly through negative effects on neurodevelopment and cognitive compensation. This distinction has evolutionary implications: common, continuously distributed, polygenic trait variation is at least compatible with long‐standing evolutionary processes, including trade‐offs, balancing mechanisms, drift, and pleiotropy, whereas rare deleterious variants are more naturally interpreted as developmental burden when they disrupt neurodevelopment or compensatory capacity (Gaugler et al. 2014; Satterstrom et al. 2020; Sella and Barton 2019; Weiner et al. 2017; Zeng et al. 2018).

A central implication is that a clinical diagnosis does not index neurodivergent trait magnitude alone. Rather, it reflects the crossing of a context‐dependent maladaptation threshold. Pronounced traits increase the likelihood of crossing this threshold. However, it may also be crossed by less pronounced trait expression when accompanied by greater burden, weaker compensatory resources, or unfavorable environmental demands. Conversely, pronounced but well‐compensated profiles may remain outside diagnostic systems and may be experienced mainly as idiosyncratic strengths, preferences, sensitivities, or self‐recognized differences. Self‐perceived difficulty is therefore not evidence that the underlying profile is intrinsically pathological; it may instead indicate decompensation of an otherwise non‐pathological trait profile under current compensatory and environmental conditions.

**FIGURE 2:**
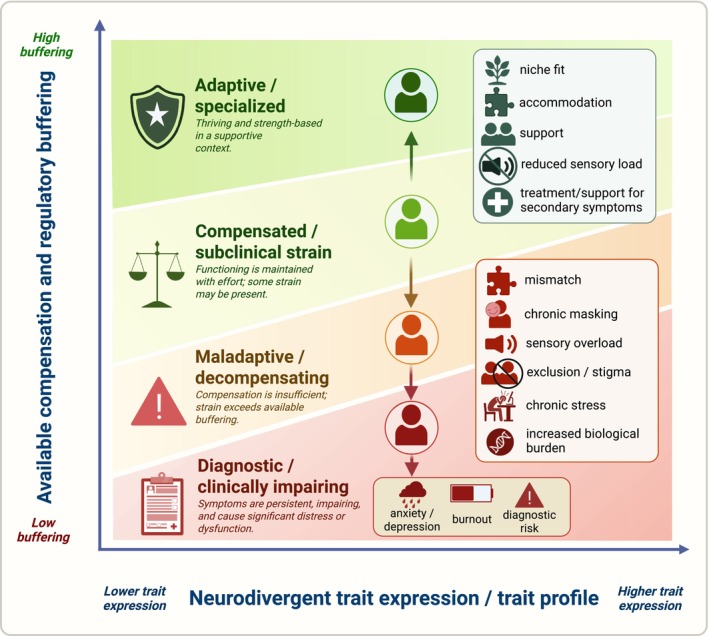
Heuristic triad‐space representation of adaptive and maladaptive clinical expression. Neurodivergent trait expression is shown on the horizontal axis, while the vertical axis represents available compensation and regulatory buffering relative to biological, developmental, and psychosocial burden. The positively sloped outcome zones reflect the threshold logic of the pathogenetic triad: When context is held constant, stronger trait expression generally requires greater compensatory and regulatory buffering to remain adaptive and compensated rather than becoming decompensating and clinically impairing. However, the same individual is not fixed to one outcome category, illustrated by the vertical trajectory. Niche fit, accommodation, social support, reduced sensory load, and treatment or support for secondary symptoms can increase effective buffering and shift expression toward adaptation. Conversely, mismatch, chronic masking, sensory overload, exclusion or stigma, chronic stress, and neuropathological or developmental insults can reduce effective buffering, shifting expression toward maladaptation. The figure is therefore a schematic clinical heuristic rather than an empirically calibrated quantitative model: The axes, slopes, zone boundaries, and trajectory are not measurement scales, estimated effects, or validated diagnostic thresholds. Empirical applications of the triad space logic require independent operationalization, measurement, and model testing. Created in BioRender; Sarovic (2026), https://BioRender.com/4g6nnsg.

Figure 2 summarizes this threshold logic schematically. It is not an empirically calibrated model: the axes, slopes, zone boundaries, and trajectory are illustrative rather than estimated from data. Its purpose is to visualize how the same underlying trait profile may be expressed differently as compensation, regulatory buffering, burden, and environmental fit change.

## 4 Beyond Trait Versus Pathology: The Triad Factors as Evolved Systems

A superficial reading of the triad might suggest that the core trait profile is the adaptive part, whereas CC and NB are merely pathological modifiers. That would be too simple. The triad is better understood as a model of interacting evolved systems and developmental constraints. This is where the framework most clearly bridges ultimate and proximate explanations: the same systems that have evolutionary histories and potential adaptive value can also generate clinical costs when expressed under unfavorable developmental, biological, or environmental conditions (Garland Jr. et al. 2022). Some components are more directly trait‐like, others more burden‐related, but none can be understood properly without considering both their evolutionary background and their proximate clinical expression.

The neurodivergent trait profile is a complex trait dimension, consistent with broader evolutionary‐genetic accounts of personality variation (Penke et al. 2007). In the original autism‐specific pathogenetic triad paper, this general factor was operationalized as AP, a continuously distributed trait dimension proposed to be influenced by common genetic variation and associated with both difficulties and strengths (Gaugler et al. 2014; Hunt and Jaeggi 2022, 2025). This framing is consistent with evolutionary accounts in which some psychopathology‐relevant trait dimensions reflect specialized cognitive profiles rather than disorder categories themselves, and with differential‐susceptibility models in which some traits may confer advantage or vulnerability depending on environmental conditions (Ellis et al. 2011). Griffin et al. (2026) develop this mismatch‐oriented argument more explicitly for depression and anxiety. In autism, traits related to precision, persistence, systemizing, and pattern detection may be advantageous in some contexts but become vulnerabilities in contexts that require rapid flexibility, tolerance of ambiguity, or inference from incomplete, implicit, or conflicting information.

CC is also a complex evolved trait, relating broadly to executive, inferential, and general cognitive resources. CC is partly heritable and genetically complex, meaning that individuals may differ in compensatory potential even in the absence of obvious developmental insult, while its expression is also shaped by developmental stability, stress exposure, learning opportunity, and biological resources (Plomin and Deary 2015). It has obvious adaptive value, but like other organismal traits it is not free of constraints or trade‐offs (Garland Jr. et al. 2022). CC should therefore be interpreted mainly as a compensatory resource, but not as a simple linear “more is always better” dimension; its ability to support compensation depends partly on profile‐environment fit. Its role is not to reduce or erase the underlying neurodivergent traits in an ontological sense, but to determine whether the consequences of those traits can be handled adaptively. In the triad, CC functions as a proximate compensatory system of adaptive capacity, while also being an evolved trait under selection and trade‐off.

NB requires more careful parsing. The term “burden” appropriately includes genuinely pathological insults, such as genetic syndromes, brain injury, infection, toxic exposure, epilepsy, or other developmental disruption. However, the systems through which burden is tolerated, amplified, or buffered are themselves evolved traits. Immune regulation and autonomic flexibility are central parts of the organism's response to developmental and environmental challenge. These systems can be understood as regulatory components of adaptive capacity, complementing the cognitive and behavioral compensation captured by CC. Immune function in particular has been strongly shaped by pathogen‐mediated selection, while modern immunoregulatory mismatch may interact with psychosocial stress to reduce stress resilience and increase inflammatory vulnerability (Quintana‐Murci 2019; Rook et al. 2013). Similarly, stress and autonomic responsivity may be adaptive when they support short‐term allostasis or rapid mobilization, but maladaptive when chronic activation produces allostatic load, persistent arousal, or inflammatory vulnerability (Garland Jr. et al. 2022; Korte et al. 2005). In this sense, immune and autonomic findings are not merely biomarkers of dysfunction, but parts of evolved regulatory systems whose costs depend on context.

This is where the triad most naturally interfaces with evolutionary psychiatry. In this model, clinical expression is hypothesized to reflect the interaction between evolved trait dimensions, adaptive capacity, developmental burden, and environmental fit. Neurodevelopmental disorder, in this view, is not the mere presence of neurodivergent variation. It is the point at which a person's profile, compensation, burden, and environment together produce persistent maladaptation to the point of fulfilling clinical criteria.

## 5 From Evolutionary Mismatch to Clinical Expression

Griffin et al. (2026) emphasize mismatch as a central explanation for elevated depression and anxiety in neurodivergent populations, but their account is broader than mismatch alone. This emphasis builds on earlier evolutionary accounts in which autistic and ADHD‐related traits were interpreted through environmental mismatch, including the possibility that traits associated with impairment in modern educational or occupational settings may have been advantageous in other ecological contexts (Swanepoel 2024; Swanepoel et al. 2017). It combines evolutionary trade‐offs and social niche specialization with the idea that affective defense systems may become chronically activated under modern conditions. This is a major contribution of Griffin et al. (2026), because it frames the neurodivergence‐environment interface through evolutionary theory. Neurodivergent traits are not treated as defects that inevitably generate psychiatric comorbidity, but as specialized profiles whose consequences depend on ecological and social fit.

This is a powerful idea, but mismatch still requires a proximate mechanism. Not every individual with a pronounced trait profile develops depression or anxiety, and the same environment does not affect all neurodivergent individuals the same way. At the individual level, mismatch becomes clinically relevant when environmental demands exceed available compensation or regulatory buffering, as illustrated in Figure 2. The difference between two individuals exposed to similar demands may instead lie in how trait expression interacts with adaptive capacity and psychosocial context. Conversely, accommodations that reduce social ambiguity, sensory load, and masking demands may shift the same individual back toward compensated or adaptive functioning. These mechanisms are exemplified in Box 2.

BOX 2 **Clinical vignette: mismatch, compensation, and decompensation**

A highly analytical autistic adult works successfully in a technical role with explicit expectations, predictable routines, written communication, low sensory load, and limited interruption. In this context, traits related to precision, persistence, pattern detection, and systemizing are functionally useful, and cognitive compensation is sufficient to manage residual social or sensory demands. The person is not clinically decompensated, and the same trait profile may be experienced mainly as a strength, preference, or cognitive style.

After organizational restructuring, the person is moved into a role requiring frequent informal meetings, rapid task‐switching, open‐plan work, ambiguous social negotiation, unpredictable interruptions, and sustained masking. The underlying trait profile has not changed, but the environment now places greater demands on compensation and regulatory buffering. Anxiety, sleep disruption, exhaustion, and depressive symptoms gradually emerge. In triad terms, this represents a shift from compensated expression toward decompensation because person–environment fit has worsened and compensatory demands have become chronically high.

The clinical implication is not that the autistic trait profile itself has become pathological, but that the same profile may have different outcomes depending on cognitive resources, regulatory buffering, accumulated burden, and environmental fit. Intervention would therefore include treating anxiety and depression when present, but also reducing avoidable mismatch through clearer expectations, lower sensory load, protected focus time, explicit communication, and reduced masking demands.

Generalizing from this example, environmental mismatch can increase the effective load placed on compensatory and burden‐buffering systems. Chronic social, sensory, and masking‐related demands may reduce available compensatory resources and increase physiological arousal. Cognitive compensation is not passive; it often involves active monitoring, inhibition, inference, and behavioral adjustment, as reflected in social camouflaging and masking strategies described in autistic adults (Hull et al. 2017; Lai et al. 2017). If such compensation must be maintained for long periods, it may become mentally draining and may contribute to exhaustion, autistic burnout, anxiety, or depression (Raymaker et al. 2020). Conversely, supportive environments may reduce burden, improve predictability, lower unnecessary cognitive load, and allow the same trait profile to be expressed more adaptively.

Taken together, this provides one plausible, testable mechanistic reading of Griffin et al.'s (2026) central argument. Evolutionary mismatch helps explain why contemporary environments may be unusually demanding for neurodivergent profiles, particularly when evolved affective and defensive systems are persistently activated under conditions of chronic stress, uncertainty, or poor fit (Nesse 2023). The triad helps explain why the consequences of those demands vary between individuals. The claim is therefore not that autism, ADHD, depression, or anxiety are non‐pathological, or that all neurodivergent presentations are adaptive. Rather, the narrower point is that evolutionarily persistent trait variation may become clinically serious when adaptive capacity is insufficient relative to environmental demand or additional burden. Depression and anxiety may also arise from primary mood or anxiety disorders, immune–inflammatory mechanisms, or other biological or psychosocial pathways (Dantzer et al. 2008; Raison and Miller 2013); the mismatch–compensation pathway is one route among several.

## 6 Reconciling Neurodiversity and the Medical Model Through Compensation and Burden

The compensation‐burden interface is where the medical model becomes necessary, but also where it must be carefully delimited. A purely neurodiversity‐based account can correctly insist that neurodivergent traits are not intrinsically pathological, but it may understate the importance of developmental burden, regulatory vulnerability, and exhausted compensation. Conversely, a purely medical model can correctly recognize impairment and suffering, but risks misattributing them to the core neurodivergent trait itself.

The pathogenetic triad offers a way to separate these levels. The relevant neurodivergent trait profile maps most naturally onto the neurodiversity model: natural cognitive variation, identity, strengths, and niche specialization. The compensation‐burden interface maps most naturally onto the medical model: maladaptation, failed compensation, and treatment need. This division is consistent with neurodiversity scholarship that frames autistic and other neurodivergent traits as part of natural cognitive variation and identity, while also allowing that disabling outcomes can arise through barriers, mismatch, and insufficient accommodation (Hunt and Procyshyn 2024). The practical consequence is that deleterious burden and exhausted or insufficient compensation should be identified, treated, prevented, or mitigated wherever possible, while the underlying trait profile should not be treated as a discrete disease lesion to be removed.

This distinction also helps reinterpret genetic and neurobiological overlap between neurodevelopmental and affective diagnoses. Such overlap need not imply that the core neurodivergent trait is itself a pathological precursor of depression or anxiety. In clinically ascertained populations, overlap may partly arise because diagnosis selects for individuals in whom neurodivergent profiles co‐occur with lower compensation, higher burden, physiological vulnerability, adverse psychosocial environments, or chronic mismatch. This point is especially important for interpreting findings that appear to support broad shared‐liability or transdiagnostic models of psychiatric comorbidity, including the general factor of psychopathology, genetic studies, and neurobiological accounts of cross‐diagnostic commonality (Bertollo et al. 2025; Caspi et al. 2014, 2024; Riglin et al. 2021). Affective symptoms in neurodivergent individuals may certainly have neurobiological correlates, and some individuals will have primary mood or anxiety disorders requiring standard psychiatric treatment. However, those correlates do not automatically prove that neurodivergence and mood disorder are expressions of a single pathological substrate. They may also reflect downstream consequences of living with a specialized profile under conditions that chronically exceed cognitive, autonomic, immune, or psychosocial buffering capacity.

This multilevel view also has implications for treatment. Evolutionarily developed trait profiles, CC, and immune‐autonomic regulation cannot be treated as if they were simple disease lesions that can be removed or corrected without consequence. Attempts to suppress or normalize neurodivergent trait expression may sometimes increase maladaptation rather than improve adaptive function. By contrast, proximate burdens conceptualized within NB should be prevented, treated, or minimized whenever possible. The clinical aim is therefore not to eliminate the neurodivergent trait profile, but to reduce avoidable burden, strengthen compensation, improve regulatory stability, treat secondary psychiatric or somatic problems when they occur, and adjust environments so that the individual has better conditions for adaptive functioning.

This distinction is also clinically important for individuals who do not meet criteria for neurodevelopmental disorders but nevertheless experience significant distress related to a pronounced trait profile. The autism‐specific pathogenetic triad paper described such cases in terms of a maladapted autistic personality, where pronounced autistic traits may contribute to secondary mental health issues without necessarily justifying a full autism diagnosis. This parallels Griffin et al.'s (2026) broader argument: neurodivergent traits are not inherently coupled to downstream psychopathology but may become associated with it when expressed under unfavorable developmental or environmental conditions.

Under this multilevel view, neurodiversity and medical models become complementary rather than mutually exclusive. Neurodiversity describes the status of the core trait as part of human variation. Medicine addresses maladaptation and suffering when trait expression, limited or exhausted compensation, burden, and environment interact unfavorably.

## 7 Toward Testable Multilevel Models

A useful multilevel framework should not only reinterpret comorbidity; it should change what can be measured and tested. Recent methodological work in evolutionary psychiatry is useful for clarifying why the integration of ultimate and proximate explanation matters empirically. Griffin et al. (2025) frame this in hypothetico–deductive terms, while Hunt and Jaeggi's (2025) DCIDE framework provides a structured approach for evaluating evolutionary hypotheses across domains of evidence. The present synthesis is complementary to this agenda because it translates ultimate claims about trade‐offs and mismatch into proximate variables, including trait profile, compensatory resources, regulatory buffering, and environmental fit, and clinical outcome. Within the triad literature, this hypothetico–deductive logic has already been applied by contrasting derived postulates between the pathogenetic triad and an alternative genomic neurodevelopmental model (Sarovic 2023), supporting falsifiability.

The distinction between heuristic representation and quantitative modeling is important in this context. Figure 2 is schematic: its axes, slopes, zone boundaries, and trajectory are illustrative rather than empirically estimated. However, the underlying triad‐space logic is intended to be operationalizable through explicit measurement of the triad components, environmental fit, and clinical outcomes. Recent quantitative work has begun to test related triad‐space formulations by modeling combinations of autistic‐trait, cognitive, and burden‐related variables in relation to clinical classification and outcome prediction (Sarovic 2026). The present figure should therefore be read as a clinical heuristic, whereas the broader framework generates empirical models that can be specified, compared, and refined.

More concretely, mismatch‐related demands such as camouflaging, sensory load, or social ambiguity should predict depressive and anxiety symptoms most strongly when neurodivergent trait expression is pronounced or uneven. Cognitive or adaptive resources should moderate these associations: greater compensatory capacity may preserve overt functioning when demands are moderate, whereas sustained compensatory effort, particularly chronic camouflaging, should predict fatigue, burnout, anxiety, or depressive symptoms when demands remain high. Autonomic or immune‐regulatory buffering should also moderate the mismatch‐symptom pathway, such that similar environmental demands are more clinically consequential when regulatory flexibility is lower or biological/developmental burden is higher.

The first methodological implication is that studies of depression and anxiety in neurodivergent populations should avoid treating autism, ADHD, or other neurodivergent profiles as homogeneous exposures. The framework instead predicts interaction effects among trait expression, adaptive capacity, environmental fit, and burden, ideally tested in longitudinal or repeated‐measures designs capable of modeling change over time.

Second, the integrated model encourages a shift from categorical comorbidity to developmental clinical reasoning. Affective symptoms in neurodivergent individuals may sometimes reflect primary mood or anxiety disorders, but they may also reflect downstream consequences of chronic overload, social exclusion, masking, sensory stress, or repeated failure to meet environmental expectations. Clinical assessment should therefore distinguish core trait features from secondary symptoms and should ask whether depression or anxiety is being generated or maintained by modifiable mismatch.

Third, the model supports prevention and accommodation. If depression and anxiety partly arise from the interaction between evolved trait profiles and mismatched environments, then intervention should not focus only on symptom reduction after disorder has emerged. It should also reduce unnecessary environmental load, increase fit between the person and the environment, strengthen compensatory resources, and prevent avoidable biological or psychosocial burden. This is relevant not only to autism but also to ADHD and other neurodevelopmental profiles, where the required adjustments may differ even if the underlying principle is the same.

Fourth, the model suggests that neurodevelopmental disorders should be studied as outcomes of interacting complex traits rather than as single disease categories. Neurodivergent trait profiles, CC, immune regulation, and autonomic function are all continuously distributed, partly heritable, and environmentally modifiable. The task is therefore not to choose between biology and neurodiversity, but to build models that can explain how biological burden and cognitive diversity interact. Empirically, this predicts interaction effects among neurodivergent trait measures, adaptive functioning, stress physiology such as heart rate variability, and depression/anxiety outcomes.

Finally, this framework underscores the need to integrate ultimate and proximate causation rather than choosing between them. Ultimate explanations can clarify why neurodivergent traits persist, why they may confer context‐dependent strengths, and why mismatch may generate vulnerability, but they do not by themselves specify the individual‐level mechanisms by which vulnerability becomes impairment. Proximate explanations can specify mechanisms of compensation, burden, and decompensation, but without evolutionary framing they may fail to explain why the relevant traits are common, stable, and sometimes advantageous. Their integration allows a more complete account: neurodivergent traits may persist because they are embedded in evolutionary trade‐offs, while clinical disorder emerges when those traits interact unfavorably with compensation, burden, and environment.

## 8 Conclusion

Griffin et al. (2026) provide an evolutionary account of why neurodivergence is so often accompanied by depression and anxiety. Their emphasis on trade‐offs, mismatch, differential environmental sensitivity, and accommodation offers a scientifically and ethically important alternative to interpreting psychiatric co‐occurrence as evidence of intrinsic defect. The pathogenetic triad complements this account by specifying a proposed cross‐level architecture through which evolved trait variation becomes clinically expressed.

This synthesis also clarifies a point already implicit in the original triad: ultimate explanations are not restricted to the core trait domain. Cognitive Capacity, autonomic regulation, and immune function are themselves evolved complex traits, shaped by selection, trade‐offs, developmental plasticity, and ecological context. Their interaction in a particular individual helps explain how evolutionary mismatch might lead to stress, exhaustion, impairment, anxiety, depression, or diagnosis.

An integrated ultimate‐proximate framework helps reconcile the neurodiversity view of natural cognitive variation with the medical reality of impairment and secondary psychiatric vulnerability. Evolutionary psychiatry clarifies why such variation may persist and why mismatch can generate vulnerability; the pathogenetic triad helps specify how that vulnerability becomes clinically significant in particular individuals.

## Funding

This work was supported by the Swedish Brain Foundation (Hjärnfonden; PS2023‐0041). The funder had no role in conceptualization, writing, or decision to submit the manuscript.

## Conflicts of Interest

The author declares no conflicts of interest.

## Data Availability

The author has nothing to report.
